# Uranine

**Published:** 1879-01

**Authors:** 


URANINE.—This is the most recently discovered, and perhaps the most remarkable, of all the coal tar or aniline group of coloring substances, now so extensively used for the adornment of the finest fabrics. Uranine is said, by chemists, to be the most highly fluorescent body known to science. Its coloring power is astonishing; a single grain will impart a marked color to nearly five hundred gallons of water.

A most interesting experiment, which anybody may try, consists in sprinkling a few atoms of Uranine upon the surface of water in a glass tumbler. Each atom immediately sends down through the water what appears to be a bright green rootlet; and the tumbler looks as if it were crowded full of beautiful plants. The rootlets now begin to enlarge, spread and combine, until we have a mass of soft green-colored liquid. Viewed by transmitted light, the color changes to a bright golden or amber hue; while a combination of green and gold will be realized, according to the position in which the glass is held. For day or evening experiment nothing can be prettier than these trials of Uranine, which are especially for the young folks: We are indebted for examples of the color to the editors of the *Scientific American*, who are sending out specimens, free of charge, to all their readers. The subscription to the paper is $3.20 for a year, or $1.60 half year; and a better investment for the money could hardly be named.

